# BALF metagenomic next-generation sequencing analysis in hematological malignancy patients with suspected pulmonary infection: clinical significance of negative results

**DOI:** 10.3389/fmed.2023.1195629

**Published:** 2023-06-28

**Authors:** Zuqun Deng, Yishu Tang, Yixuan Tu, Mei Liu, Qian Cheng, Jian Zhang, Feiyang Liu, Xin Li

**Affiliations:** ^1^Department of Hematology, The Third Xiangya Hospital, Central South University, Changsha, Hunan, China; ^2^Department of Emergency, The Third Xiangya Hospital, Central South University, Changsha, Hunan, China

**Keywords:** metagenomic next-generation sequencing, pulmonary infection, bronchoalveolar lavage fluid, pathogen, diagnosis

## Abstract

**Purpose:**

Metagenomic next-generation sequencing (mNGS) of bronchoalveolar lavage fluid (BALF) is gradually being used in hematological malignancy (HM) patients with suspected pulmonary infections. However, negative results are common and the clinical value and interpretation of such results in this patient population require further analysis.

**Methods:**

Retrospective analysis of 112 HM patients with suspected pulmonary infection who underwent BALF mNGS and conventional microbiological tests. The final diagnosis, imaging findings, laboratory results and treatment regimen of 29 mNGS-negative patients were mainly analyzed.

**Results:**

A total of 83 mNGS positive and 29 negative patients (15 true-negatives and 14 false-negatives) were included in the study. Compared to false-negative patients, true-negative patients showed more thickening of interlobular septa on imaging (*p* < 0.05); fewer true-negative patients had acute respiratory symptoms such as coughing or sputum production (*p* < 0.05) clinically; On the aspect of etiology, drug-related interstitial pneumonia (6/15, 40%) was the most common type of lung lesion in true-negative patients; on the aspect of pathogenesis, false-negative patients mainly missed atypical pathogens such as fungi and tuberculosis (8/14, 57.1%). Regarding treatment, delayed anti-infection treatment occurred after pathogen missing in mNGS false-negative patients, with the longest median time delay observed for anti-tuberculosis therapy (13 days), followed by antifungal therapy (7 days), and antibacterial therapy (1.5 days); the delay in anti-tuberculosis therapy was significantly longer than that in antibacterial therapy (*p* < 0.05).

**Conclusion:**

For HMs patients with imaging showing thickening of interlobular septa and no obvious acute respiratory symptoms, lung lesions are more likely caused by drug treatment or the underlying disease, so caution should be exercised when performing BALF mNGS. If BALF mNGS is negative but infection is still suspected, atypical pathogenic infections should be considered.

## 1. Introduction

Pulmonary infections are commonly observed in hematologic malignancy (HM) patients. Roughly 20–50% of HM patients will suffer from pulmonary infections during treatment (1, 2), leading to future complications and poor prognosis (3, 4); the mortality rate of HM patients with pneumonia for example is as high as 80% (3). For HM patients with low immune functionality and high susceptibility to pulmonary infections, timely identification of pathogens and administration of correct medications can greatly improve patient outcomes (5, 6).

Metagenomics next generation sequencing (mNGS) is a rapid and comprehensive method of pathogen detection. The diagnostic efficacy of mNGS for immunocompromised patients with pulmonary infections has been demonstrated in prior research (7, 8). A positive mNGS result derived from alveolar lavage fluid obtained through fiberoptic bronchoscopy can assist clinicians in identifying pathogens responsible for pulmonary infections and help clinicians to subsequently adjust antibiotic usage and treatment regimens. However, HM patients commonly suffer from cytopenia and abnormal coagulation. For these patients, employing fiberoptic bronchoscopy to obtain alveolar fluid for mNGS analysis can bring increased risks (9). Due to these risks, clinicians must consider what kind of patients can undergo bronchoalveolar lavage fluid (BALF) mNGS analysis. In addition, clinicians must consider how to effectively interpret negative mNGS results and consider whether such results are due to a technical misdiagnosis or a genuine absence of pulmonary infection. Current research discussing the interpretation of negative mNGS results is lacking, despite wide usage of mNGS analysis in HM patients. Understanding and developing strategies to analyze negative mNGS results can be beneficial by allowing patients to avoid unnecessary risks, saving costs on unneeded examinations, and helping clinicians accurately prescribe medications.

This study aims to address the aforementioned research gap through a detailed analysis of clinical data drawn from HM patients with suspected pulmonary infection. This study will focus particularly on the analysis of mNGS negative patients, describing the characteristics of such patients, determining the most appropriate circumstances to perform a BALF mNGS analysis, and discussing optimal approaches to deal with mNGS negative HM patients with suspected pulmonary infection.

## 2. Materials and methods

### 2.1. Study designs

This retrospective study looked at patients from the 3rd Xiangya Hospital of Central South University in China who were suspected to have had pulmonary infections between April 2019 and June 2022. Patients were eligible for enrollment if they met the following criteria: (1) ≥16 years of age; (2) Diagnosed with hematological malignancy; (3) Patients had a suspected pulmonary infection and bronchoalveolar lavage fluid (BALF) was subsequently obtained for mNGS.

Patients were suspected of having a pulmonary infection if the following inclusion criteria were met: a new patchy or progressive infiltrate, consolidation, ground-glass opacity, pleural effusions, micronodules, or nodules observed in imaging, and one of the following four symptoms: (1) Fever; (2) Cough, expectoration, hypoxia or aggravation of existing respiratory symptoms; (3) Peripheral blood white blood cell (WBC) count > 10 × 10^9^/l or < 4 × 10^9^/L; (4) Clinical signs of pulmonary consolidation or wet rales.

Patients were excluded if their medical records were incomplete or lacked paired conventional microbiological tests (CMTs). This study was carried out in accordance with the principles of the Declaration of Helsinki and was approved by the Ethics Review Committee of the 3rd Xiangya Hospital of Central South University (No:23049). Patient approval and written informed consent were waived due to the study’s retrospective design.

### 2.2. BALF mNGS

Bronchoscopies were performed using a flexible fiberoptic bronchoscope (Olympus). Procedures were performed through the nasal or oral cavity after sedation (5 mg intravenous midazolam) and local anesthesia (lidocaine 2%) and were supplemented with cardiopulmonary monitoring. Patients with a platelet counts below 20 × 10^9^/L received 4 units of platelets within 1 h prior to the procedure. BALF was obtained from patients using standard methods. The collected BALF samples were divided into two groups and sent to the laboratory for mNGS and CMTs.

Metagenomic next-generation sequencing procedures included specimen pretreatment, nucleic acid extraction, library construction and sequencing. If an RNA viral infection was suspected, RNA extraction and sequencing procedures were conducted simultaneously. Following the manufacturer’s instructions, DNA was extracted using a QIAamp UCP Pathogen DNA Kit (Qiagen). Benzonase (Qiagen) and Tween20 (Sigma) were used to extract human DNA (10). QIAamp Viral RNA Kit (Qiagen) was used to extract total RNA, and a Ribo-Zero rRNA Removal Kit was used to remove ribosomal RNA (Illumina). Reverse transcriptase and dNTPs (Thermo Fisher) were used to create cDNA. A Nextera XT DNA Library Prep Kit (Illumina, San Diego, CA, USA) was used to create libraries for the DNA and cDNA samples. The library’s quality was controlled by Qubit dsDNA HS Assay Kit and High Sensitivity DNA kit (Agilent) on an Agilent 2100 Bioanalyzer. Each library pool was then loaded onto an Illumina Nextseq 550Dx sequencer for sequencing (11), In parallel with each batch, we prepared PBMC samples with 10^5^ cells/mL from healthy donors using the same methodology, and sterile deionized water was extracted alongside the specimens to use as non-template controls (NTC).

### 2.3. BALF mNGS results analysis

Using Trimmomatic to remove low quality reads, adapter contamination, and duplicated reads, as well as those shorter than 50 bp (12). Human sequence data were identified by mapping to a human reference (hg38) using Burrows-Wheeler Aligner software and then excluded (13). The remaining sequence data were aligned to the current databases (NCBI).^1^ The pathogen lists were chosen based on three sources: (1) Johns Hopkins ABX Guide^2^ (2) Manual of Clinical Microbiology^3^ (3) clinical case reports or research articles published in current peer-reviewed journals. Virus-positive detection results (DNA or RNA viruses) were defined by coverage of three or more non-overlapping regions in the genome. A positive detection was reported for a given species or genus if the reads per million (RPM) ratio, or RPM-r was ≥10, (RPM-r:RPM (sample)/RPM (NCT), the RPM corresponding to a given species or genus in the clinical sample divided by the RPM in the NTC).

Microorganisms detected by mNGS which met all the criteria were identified as suspected pathogens. Due to discrepancies between bioinformatics analysis and clinical reality, in our study, although Torque teno virus was detected as a suspected pathogen based on bioinformatics analysis, clinicians considered the virus not pathogenic; mNGS results which detected only Torque teno virus were therefore categorized as negative, True-negative patients were defined as patients with a negative mNGS test and a final diagnosis of non-pulmonary infection. False-negative patients were defined as patients with a negative mNGS test and a genuine pulmonary infection.

### 2.4. Conventional microbiological tests

All patients received conventional microbiological tests (CMTs) for identifying pathogens based on the patient’s condition during hospitalization. The choice of conventional microbiological tests was decided by doctors, The most commonly used conventional diagnostic methods for microbial diagnosis are bacterial and fungal culture, real-time polymerase chain reaction (PCR) was used to detect cytomegalovirus (CMV), Epstein–Barr virus (EBV). the remaining BALF samples was used for culture and smear microscopy to identify pathogens and also assessed by galactomannan (GM) tests. The detection of fungi also includes GM and (1/3)-β-D-glucan tests in blood. Enzyme-linked immunospot assay (T-SPOT). GeneXpert mycobacterium tuberculosis (MTB) and TB-DNA test were preformed for patients with suspected tuberculosis. Specimen types include throat swabs, sputum, bronchoalveolar lavage fluid (BALF), blood and pleural effusion. Swallow swabs are only used to detect respiratory viruses and atypical pathogens by PCR (including Influenza A/B Viruses, Adenovirus, Respiratory Syncytial Virus, Rhinovirus, COVID-19, Chlamydia, and Mycoplasma), conventional diagnostic methods can be repeated if necessary.

### 2.5. Diagnostic criteria for pneumonia

Two senior hematologists conducted a comprehensive analysis of the patient’s clinical features, imaging data, conventional microbiological tests, mNGS results, and treatment results to determine whether the patient had pulmonary infection and further determine the pathogens that cause infectious diseases. We also have invited experts in the fields of radiology and microbiology to assist in the interpretation of the results. For cases that cannot be diagnosed, another senior clinician will be consulted.

Invasive fungal disease was defined according to the European Organization for Research and Treatment of Cancer and the Mycoses Study Group Education and Research Consortium (EORTC/MSGERC) criteria (14). For “Probable” Invasive pulmonary fungal infections, imaging experts and clinicians of our hospital are required to make diagnoses together.

### 2.6. Statistical analysis

Analyses were performed using IBM SPSS Statistics 26.0 and Graphpad Prism 9.3.0. Continuous variables were expressed as mean ± standard deviation or the median ± quartile spacing. Categorical variables were expressed as numbers and percentages. For continuous variables, Student’s *t*-tests were used to compare differences between groups if the variables were normally distributed, and non-parametric tests were used when variables were non-normally distributed. The chi-squared test or Fisher’s exact test were used for categorical variables. All tests were two-tailed. *P*-values < 0.05 were considered significant.

## 3. Results

### 3.1. Patient characteristics

A total of 112 HM patient records were included in the analysis (Supplementary Table 1), with 83 mNGS positive-patients and 29 mNGS negative-patients. Following final diagnosis, 29 mNGS negative patients were divided into two groups: true-negative (*n* = 15, final diagnosis determined patients did not have pulmonary infection), and false-negative (*n* = 14, final diagnosis determined patients had pulmonary infection). Comparison of the three patient groups (true-negative, false-negative, positive) revealed significant differences in clinical and imaging features of the true negative group, compared with the other two groups. In terms of radiological features, the proportion of true-negative patients with interstitial thickening was higher than that of false-negative patients (33.3% vs. 0%, *P* < 0.05) and positive patients (33.3% vs. 7.2%, *P* < 0.05). In terms of clinical manifestations, the proportion of true-negative patients with fever was lower than that of positive patients (20.0% vs. 59.0%, *P* < 0.05), while the proportion of true-negative patients with cough and expectoration (26.7% vs. 92.9%, *P* < 0.05) was lower than that of false-negative patients. The remaining characteristics of underlying disease, disease status, and infection indicators did not show significant differences between the three groups (Table 1). Therefore, true-negative patients exhibited more specific radiological changes in the lungs, but had fewer acute respiratory symptoms.

**TABLE 1:** Clinical characteristics of 112 patients with HM disease.

Variables	mNGS true-negative n (%)	mNGS false-negative (%)	**^a^*p*	mNGS positive n (%)	**^b^*P*
	**(N = 15)**	**(N = 14)**		**(N = 83)**	
**Demographic information**					
Male sex	9 (60.0)	7 (50.0)	0.715	52 (62.7)	0.845
Age (y), mean ± SD	51.00 ± 10.744	43.93 ± 14.510	0.145	48.5 ± 14.656	0.678
**Underlying diseases**					
AML	5 (33.3)	5 (35.7)	1.000	29 (34.9)	0.904
ALL	1 (6.7)	4 (28.6)	0.169	14 (16.9)	0.535
NHL	3 (20)	3 (21.4)	1.000	18 (21.7)	1.000
**Disease status**					
Complete remission	9 (60.0)	8 (57.1)	1.000	54 (65.1)	0.707
Previous HSCT or CAR T-cells therapy	3 (20)	5 (35.7)	0.426	23 (27.7)	0.761
**Laboratory parameters**					
WBC, 10^9^/L	3.92 ± 2.19	4.80 ± 5.31	0.559	6.66 ± 7.82	0.214
ANC, 10^9^/L	2.45 ± 1.68	2.89 ± 4.16	0.708	4.03 ± 4.50	0.339
PCT ≥ 0.25 ng/mL	2 (13.3)	2 (14.3)	1.000	24 (28.9)	1.000
CRP ≥ 5 mg/L	8 (53.3)	6 (42.9)	0.715	39 (47.0)	0.651
**Abnormality on chest radiograph**					
Unilateral lesion	2 (13.3)	7 (50.0)	0.050	13 (15.7)	1.000
Nodule	9 (60.0)	8 (57.1)	1.000	32 (38.6)	0.121
GGO	6 (40.0)	4 (28.6)	0.700	35 (42.2)	0.847
Interlobular septal thickening	5 (33.3)	0 (0.0)	0.042*	6 (7.2)	0.012*
Hilar or mediastinal LN enlargement	4 (26.7)	4 (28.6)	1.000	18 (21.7)	0.929
Pleural effusion	3 (20.0)	3 (21.4)	1.000	15 (18.1)	0.859
**Clinical symptoms**					
Fever	3 (20.0)	8 (57.1)	0.060	49 (59.0)	*0.005* **
Cough	4 (26.7)	13 (92.8)	*0.001* ***	48 (57.8)	*0.026*
Previous antibiotic exposure	10 (66.7)	13 (92.8)	0.169	82 (98.8)	*0.001* ***
Length of hospitalization	31.13 ± 18.73	24.00 ± 22.70	0.363	25.39 ± 17.91	0.258

AML, acute myeloid leukemia; NHL, non-Hodgkin’s lymphoma; ALL, acute lymphoblastic leukemia; HSCT, hematopoietic stem-cell transplantation; CAR, chimeric antigen receptor; GGO, ground-glass opacity; LN, lymph node.

^a^*p*:*p*-values for mNGS true-negative and mNGS false-negative.

^b^*p*:*p*-values for mNGS true-negative and mNGS positive.

*p < 0.05; **P < 0.01; ***P < 0.001.

### 3.2. Analysis of true-negative patients

#### 3.2.1. Diagnosis of true-negative patients

**FIGURE 1:**
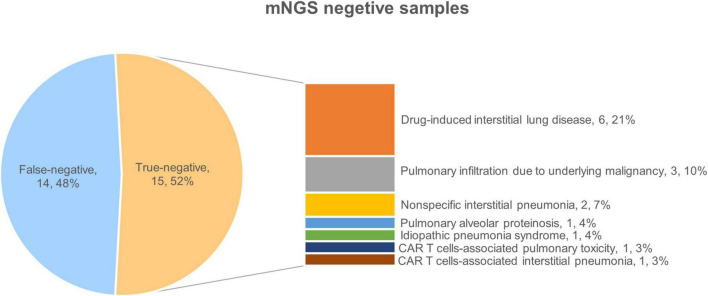
Final diagnoses of true-negative group patients.

Further analysis was conducted on the clinical data of the 15 patients who tested true negative. Of these, 10 patients ultimately received a diagnosis of interstitial pneumonia, Among them, there were 6 patients of drug-induced interstitial lung disease (DI-ILD), 2 patients of non-specific interstitial pneumonia, 1 patient of immunologically related interstitial pneumonia that occurred in the late stage after chimeric antigen receptor T cell (CAR-T) therapy, and 1 patient of secondary pulmonary alveolar proteinosis (PAP). Among the remaining 5 patients, 3 patients were diagnosed with pulmonary infiltration due to underlying malignancy, 1 patient was diagnosed with pulmonary toxicity caused by CAR T-cells’ early cytokine release syndrome (P: patient, P18), and 1 patient was diagnosed with idiopathic pneumonia syndrome (IPS) after transplantation (P2) (Figure 1). Patients presented with unique clinical features, related to patients’ primary disease. The 10 patients receiving a final diagnosis of interstitial pneumonia had all been previously treated with antineoplastic drugs (P4, P7, P10, P12, P13, P14, P20, P27, P28, and P29). The 3 patients with pulmonary infiltration due to underlying malignancy had uncontrolled primary disease (P5, P17, and P24). The remaining two patients received specific treatments (transplantation and CAR T-cells therapy).

Overall, all true-negative patients had pulmonary imaging changes, the pulmonary changes in mNGS-negative HM patients are closely related to the underlying diseases and treatments, with DI-ILD being the most common.

#### 3.2.2. Diagnosis of true-negative patients

**FIGURE 2:**
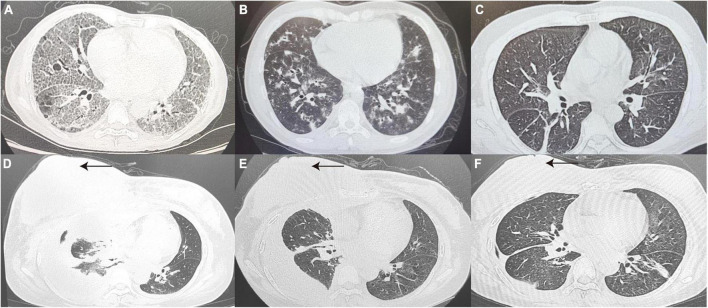
True-negative representative cases. **(A)** A 41 year old woman diagnosed with pulmonary alveolar proteinosis, CT Scan revealed thickened interlobular septa and multiple patchy with a reticular pattern changes in the subpleural of Bilateral lungs. **(B)** A 32 year old post-transplant AML patient with a final diagnosis of IPS, CT Scan revealed diffuse distribution of flocculent hyperdense shadows in Bilateral lungs, with some lesions fused into a sheet. **(C)** Chest CT Images confirmed drug-induced interstitial lung disease, showing interstitial changes in Bilateral lungs with disorganized broncho-vascular bundles and interlobular septal thickening with a reticular pattern. **(D–F)** Patient with relapsed DLBCL presented with chest tightness and pleural effusion on CT, 7 days after infusion of anti-CD19 CAR T-cells **(D)**. At the same time, patient’s peripheral blood CAR T-cell copy number, IL-6 and ferritin were elevated, consideration of CAR T-cell-associated pulmonary toxicity, patient lung lesions improved 1 month after tolimumab and chest fluid drainage **(E)**. A review more than 3 months later showed right breast lesion (as indicated by the black arrow) and lung lesion were significantly better than before **(F)**. CT, computed tomography; AML, acute myeloid leukemia; DLBCL, diffuse large B-cell lymphoma; IL-6, interleukin-6.

In terms of radiological features, 33.3% of patients showed more specific changes in interstitial thickening (P4, P10, P14, P29, and P18). Figure 2 presents the imaging of some representative true-negative patients.

#### 3.2.3. Treatment and specific information table of true-negative patients

In terms of treatment, 5 (33.3%) patients had their antibiotics reduced and stopped after mNGS results were reported (P4, P17, P24, P27, and P29). Based on the etiology of lung lesions and a negative mNGS result, 66.7% of true negative patients adjusted anti-tumor or immunosuppressive agent dosing in accordance with their conditions; adjustments included adding hormones, chemotherapy, tocilizumab or anti-rejection drugs (P2, P5, P10, P7, P12, P24, P28, and P18). Some patients with drug-related pulmonary lesions stopped taking relevant medications after further consideration of risks and benefits (P13, P14). Partial negative mNGS results can assist clinicians in ruling out infectious etiologies. The patients’ final diagnosis was determined by examining patient specific medical history and analyzing relevant changes before and after treatment. Information and diagnoses of the 15 true negative patients are outlined in Table 2.

**TABLE 2:** The true-negative mNGS results and clinical diagnosis analysis in 15 patients.

NO	Age/Sex	Underlying medical conditions	Clinical manifestations	Final diagnosis	Assessment and treatment plan	Main lung imaging findings
2	32/M	AML	Cough, Sputum	Idiopathic pneumonia syndrome	1. 120 days post-transplant 2. Reduce/discontinue ineffective antibiotic treatment. Improvement after treatment with methylprednisolone, ruxolitinib, tacrolimus.	Diffuse distribution of flocculent hyperdense opacity in bilateral lungs
4	41/F	MDS	Shortness of Breath	Pulmonary alveolar proteinosis	1. Lung Biopsy PAS ( + ), potential pulmonary alveolar proteinosis. 2. Discontinue/reduce ineffective antibiotic treatment. Improvement after treatment with GM-CSF, cyclosporine, and methylprednisolone.	Thickening of interlobular septa, multiple lamellar faint shadows seen under pleura, reticular pattern
5	65/M	Plasma Cell Leukemia	Cough, Sputum, Fever	Leukemic pulmonary infiltration	1. Relapsing disease with multiple sites of extramedullary malignant plasma cell infiltration. 2. Antibiotic treatment ineffective. Improvement after chemotherapy and BCMA-CAR T-cells infusion.	Right lung ground glass opacity; small nodular opacity in both lungs; enlarged lymph nodes in mediastinum, sternal stalk leukemia bone destruction
7	54/M	AML	N/A	Non-specific interstitial pneumonia	1. Idarubicin + cytarabine regimen chemotherapy 3 times, not associated with drug therapy, no evidence of infection 2. No antibiotics or hormones, no pathological changes post-transplant.	Ground glass opacity observed in basal segment of anterolateral right lung
10	46/M	NHL	Occasional Shortness of Breath	Drug-induced interstitial lung disease (R-CHOP)	1. Six prior chemotherapy sessions (R-CHOP) 2. No improvement post anti-infection treatment, CART infusion considered, no hormone therapy.	Thickening of interlobular septa, reticular pattern
12	58/F	ALL	N/A	Non-specific Interstitial Pneumonia	1. Previous chemotherapy with VDCLP, CAM regimen. No evidence of infection, no significant drug interaction 2. No antibiotic treatment, no pathological change after transplantation	Patchy and striated hyperdense images seen in bilateral lungs
13	42/M	CML	N/A	Drug-induced interstitial lung disease (Ponatinib)	1. Observed after 1 month treatment with ponatinib 2. No antibiotics, improved after stopping ponatinib	Diffuse distribution of small nodules in bilateral lungs
14	71/M	MM	Shortness of Breath	Drug-induced interstitial lung disease (Pomalidomide)	1. Observed after treatment with pomalidomide 2. Antibiotic treatment ineffective, improvement observed after discontinuing pomalidomide	Multiple small patchy faint density shadows with mosaic changes both lungs, thickening of interlobular septa
17	47/F	AML	Cough, Sputum, Fever	Leukemic pulmonary infiltration	1. Initial diagnosis of leukemia, leukocytes greater than 50*109/L on admission to hospital. 2. Multiple antibiotic treatment ineffective, improvement after chemotherapy.	Small ground glass nodule foci in the right lung
18	49/F	NHL	Cough, Sputum, Fever	CAR T-cells-associated pulmonary toxicity	1.3 days post CAR T-cells treatment, CAR T-cells copy number, IL-6 elevated 2. Antibiotic treatment ineffective, improvement after oxygen, pleural fluid drainage, and tolzumab.	Massive right pleural effusion, Right lung atelectasis, Consolidation, Thickening of interlobular septa, soft tissue foci on right posterior segment of right sternum larger than before
20	39/F	MM	N/A	CAR T-cells-associated interstitial pneumonia	1.164 days post CAR T-cells treatment, CAR T-cells copy number, IL-6 elevated 2. Antibiotic treatment ineffective, improvement after oxygen and nebulization.	Patches in both lungs, flocculent high density opacity, Ground Glass Opacity, subpleural line seen in lower lobe of right lung
24	61/M	MM	Shortness of Breath	Myelomatous pulmonary infiltrates	1. Initial diagnosis of myeloma with abnormal plasma cells seen in BALF. 2. No improvement after anti-infection treatment, improvement observed after chemotherapy.	Multiple patches of ground glass density and striated high density opacity in bilateral lungs
27	50/F	NHL	Occasional Shortness of Breath	Drug-induced interstitial lung disease (R-CHOP)	1. Previously treated with five R-CHOP chemotherapy sessions. 2. Anti-infection treatment ineffective and discontinued, improvement observed after adding methylprednisolone.	Dorsal segment of lower lobe of right and lower lobe of left lung show fibrous stripes
28	48/M	AML	Occasional Shortness of Breath	Drug-induced interstitial lung disease (Cytarabine)	1. Previous chemotherapy with idarubicin + cytarabine regimen 1 time, high dose cytarabine chemotherapy 3 times. 2. Not treated with antibiotics, no infection observed	Cystic thin-walled translucent shadow in left lung, nodular hyperdensity shadow
29	62/M	AML	Shortness of Breath	Drug-induced interstitial lung disease(Azacitidine)	1. One prior chemotherapy with Azacitidine + Vinecla, decreased diffusion function in the lungs. 2 Antibiotic treatment ineffective and stopped, improvement after adding methylprednisolone.	Thickening of interlobular septa in bilateral lungs, localized honeycombing like changes

GM-CSF, granulocyte-macrophage colony-stimulating factor; R-CHOP, rituximab, cyclophosphamide, Doxorubicin, Vincristine and Prednisolone immunochemotherapy; VDCLP, vincristine, daunomycin, cyclophosphamide, asparaginase and dexamethasone immunochemotherapy; CAM, cyclophosphamide, doxorubicin, and methotrexate immunochemotherapy; PAS, periodic acid-schiff; MDS, myelodysplastic syndromes.

### 3.3. Analysis of false-negative patients

#### 3.3.1. Diagnosis of false-negative patients

**FIGURE 3:**
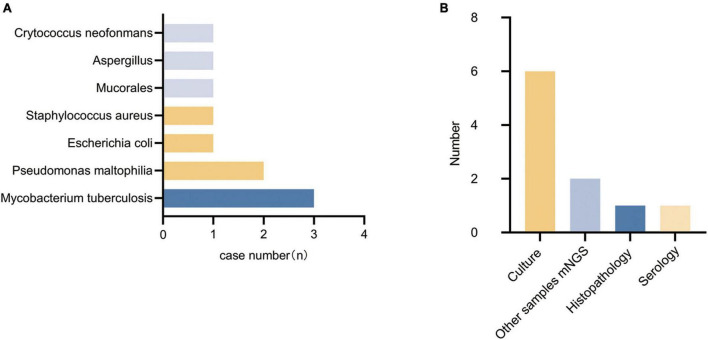
Types of pathogens detected in the false-negative group and methods of detection. **(A)** Types of pathogens detected in the false-negative group. **(B)** Methods of detection.

A total of 14 patients had their pathogens missed by mNGS, including 5 patients of fungal pneumonia, 3 patients of tuberculosis, 4 patients of bacterial pneumonia (28.6%), and 2 patients (14.3%) with unknown pathogens. Among the missed fungi, 2 cases were detected by sputum culture (P9, P23), 1 case was diagnosed as positive for serum capsule antigen (P21), and 2 cases were clinically diagnosed as fungal pneumonia (P6, P22). Of the missed tuberculosis cases, 2 were detected in other samples by mNGS (P8, P25), and 1 case was diagnosed with pulmonary tuberculosis by lung tissue pathology biopsy (P15). The missed bacteria (P1, P16, P19, and P26) were all detected by sputum or lavage fluid culture. Two patients with pulmonary infections (P6, P11) did not have clear pathogenic evidence, but their conditions improved after empirical antimicrobial therapy. Detected pathogens are shown in Figure 3A, specific methods of detection are shown in Figure 3B.

The missed pathogens in these patients were mainly atypical pathogens (57.1%) (fungi and tuberculosis).

#### 3.3.2. Diagnosis of false-negative patients

**FIGURE 4:**
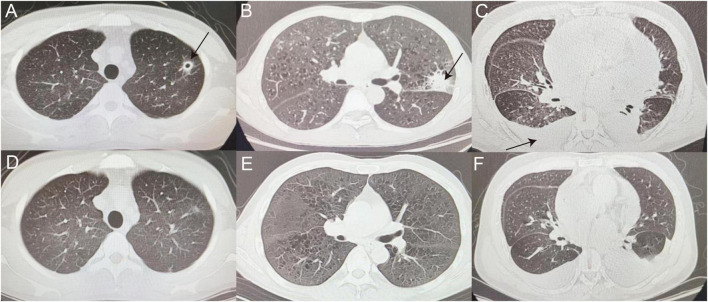
False-negative representative cases. **(A,D)** CT image of man with thin-walled cavity in the upper lobe of left lung (as indicated by the black arrow), eventually diagnosed with cryptococcal pneumonia, before **(A)** and after **(D)** voriconazole treatment. **(B,E)** Left tuberculoma shadow (as indicated by the black arrow) observed in patient with ALL **(B)**, referred to tuberculosis hospital for anti-tuberculosis treatment and improved **(E)**. **(C,F)** Patient with MDS with recurrent cough and fever during chemotherapy, with scattered multiple patchy fuzzy shadows in Bilateral lungs, incomplete expansion of the lower lobes of Bilateral lungs, and pleural effusion (as indicated by the black arrow) **(C)** was sent for tuberculosis diagnosis after mNGS of pleural fluid and transferred to hospital for anti-tuberculosis treatment **(F)**. ALL, acute lymphoblastic leukemia; MDS, myelodysplastic syndromes.

After confirmed diagnosis, false-negative patients showed improvement after receiving anti-infection treatment, as demonstrated by the imaging in Figure 4 which represents some typical cases.

#### 3.3.3. Treatment and specific information table of false-negative patients

**FIGURE 5:**
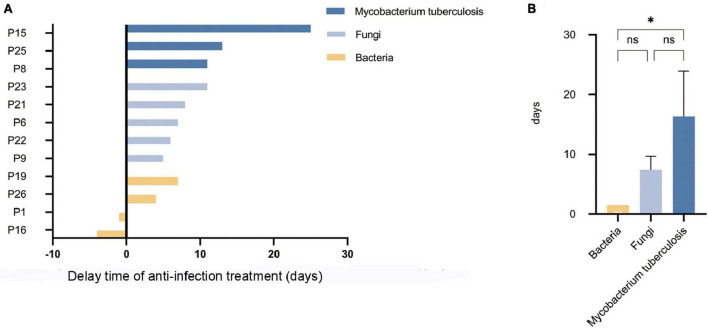
The delay time of anti-infection treatment caused by missed pathogens detected by mNGS. **(A)** The delay time of anti-infection treatment caused by missed pathogens detected by mNGS. **(B)** The delay time for anti-tuberculosis treatment was much higher than that for anti-bacterial treatment (*p < 0.05; ns. not statically significant).

In terms of treatment, previously inappropriately administered antibiotics were discontinued in 5 (35.7%) false-negative patients (P1, P15, P19, P25, P26) after final diagnosis was clarified. Eventually appropriate antibiotics were provided to all (100.0%) patients, However, most patients experienced delayed appropriate anti-infection treatment due to failure in timely pathogen detection by mNGS. The delay time of anti-infection treatment caused by missed pathogens detected by mNGS is shown in Figure 5A. Among them, the median delay time was 13 days for anti-tuberculosis treatment, 7 days for anti-fungal treatment, and 1.5 days for anti-bacterial treatment. The delay time for anti-tuberculosis treatment was much higher than that for anti-bacterial treatment, and the difference was statistically significant (*p* < 0.05), as shown in Figure 5B.

In conclusion, false-negative patients missed mainly atypical pathogens, and the delays in treatment for patients with missed tuberculosis were significantly longer than those for missed bacterial infections. Details of all false-negative patients, their final diagnosis, and diagnostic basis can be found in Table 3.

**TABLE 3:** The false-negative mNGS results and clinical diagnosis analysis in 14 patients.

NO	Age/Sex	Underlying medical conditions	Clinical manifestations	Final diagnosis	Assessment and treatment plan	Main lung imaging findings
1	50/M	NHL	Fever, cough, shortness of breath	Staphylococcus aureus pneumonia	1. Sputum culture: Staphylococcus aureus, presence of abscess, pneumothorax, and other manifestations 2. Meropenem decreased to cefoperazone, improvement observed with addition of anti-gram positive teicoplanin	Solid left lung atelectasis, with left pleural encapsulated effusion
3	22/F	ALL	Fever, cough	Pulmonary infection (pathogenesis unknown)	1. Improvement after anti-infection treatment	Multiple cords and patches of hyperdensity in Bilateral lungs.
6	46/F	AML	Cough, sputum, fever	Fungal pneumonia	1. presence of risk factors for fungal infection and secondary clinical features 24 days post transplantation 2. Antibacterial and antiviral treatments were ineffective, improvement observed with voriconazole antifungal treatment.	New streaky hyperdensity shadow in the right lung
8	31/F	AML	Cough, sputum	Secondary Tuberculosis	1. mNGS of liver tissue suggests Mycobacterium tuberculosis, presence of pulmonary and extrapulmonary tuberculosis 2. Symptoms improved after anti-tuberculosis treatment	New streaky hyperdensity shadow in the right lung, scattered small nodular foci in bilateral lungs as before, multiple lymph nodes in mediastinum as before
9	38/F	ALL	Cough, Sputum	Pulmonary aspergillosis	1.Sputum culture: Aspergillus, 28 days after transplantation 2.Improvement after voriconazole antifungal treatment	Multiple nodules and patchy foci of ground glass density were seen in bilateral lungs, with the lower lobe being the most prominent. Solid nodule foci seen on the right.
11	57/F	AML	Cough, Sputum	Pulmonary infection, (pathogenesis unknown)	1.Improvement observed after anti-infection treatment.	Multiple patchy and blurred shadows in the lower lobes of bilateral lungs.
15	55/M	ALL	Cough, Fever, difficulty breathing	Secondary Tuberculosis	1. TSPOT: + , lung tissue biopsy, positive antacid staining 2. Improved after transferring to hospital for anti-tuberculosis treatment	Patchy ground glass hyperdensity in the upper lobe of the left lung.
16	57/F	ALL	Cough, sputum	Escherichia coli pneumonia	1. Sputum culture: Escherichia coli 2. Improvement after piperacillin treatment	Scattered multifocal foci of faint ground glass density in bilateral lungs, solid shadow in the middle lobe of the right lung
19	52/F	AML	Cough, Fever, shortness of breath	Pseudomonas maltophilia pneumonia	1. Sputum culture: Pseudomonas maltophilia 2. No improvement with meropenem, improvement with levofloxacin treatment	Scattered multiple small patchy hyperdense shadows in bilateral lungs, patchy ground glass opacities, small ground glass nodules
21	26/M	EBV + LPD	fever	Cryptococcal pneumonia	1. Slightly higher G-lab and cavity formation in the lungs 1 year after transplantation 2. Improvement after antifungal treatment with voriconazole.	Thin-walled cavity in the upper lobe of the left lung
22	52/M	NHL	Cough, Shortness of breath, difficulty breathing	Fungal pneumonia	1. Bronchial brush examination excludes lung cancer, fungal infection considered upon observing granular material microscopically 2. Solid wedge-shaped lesion was seen on imaging, improved after voriconazole treatment	Large solid foci in the upper lobe of the left lung.
23	57/M	AML	Fever, cough, sputum	Pulmonary Mucormycosis	1. Sputum culture showed growth of Trichoderma spp. 2. Voriconazole treatment ineffective, improvement after amphotericin B treatment	A nodular and mass like high density shadow was seen in the right lung. In the left lung, a blurred mass like high density shadow was also seen in the posterior basal segment of the lower lobe
25	56/M	MDS	Cough, fever	Secondary Tuberculosis	1. TSPOT: + , pleural fluid mNGS: Mycobacterium tuberculosis. 2. Antifungal treatment ineffective, patient transferred to anti-tuberculosis treatment to improve	Scattered multilamellar fuzzy shadows in bilateral lungs, incomplete expansion of bilateral lungs, pleural effusion
26	16/M	NHL	Cough, sputum	Pseudomonas maltophilia pneumonia	1. Alveolar lavage fluid culture: Pseudomonas maltophilia 2. No improvement with meropenem, improvement with levofloxacin treatment	Left lung nodular hyperdensity shadow

EBV + LPD Epstein-Barr virus + lymphoproliferative disorders.

## 4. Discussion

Hematological malignancy patients have a high incidence of pulmonary infections and obtaining timely pathogenic evidence and correctly using antibiotics can greatly improve patient prognosis. mNGS, a comprehensive and rapid but invasive and relatively expensive test, is increasingly being used in the pathogenic diagnosis of HM patients with suspected pulmonary infections (9). It is crucial to reduce unnecessary mNGS testing and properly analyze and treat negative mNGS results after they occur. Our study is the first report analyzing negative mNGS results in HM patients with suspected pulmonary infections.

The analysis of true negative patients revealed that the characteristics of non-infectious pulmonary lesions in HM patients are closely related to the HM disease itself and its associated treatment. The non-infectious etiologies of pulmonary lesions in HM patients include pulmonary changes caused by chemotherapeutic drugs, pulmonary infiltration from the HM disease, and targeted immunosuppressive drugs. Imaging showed the non-infectious etiology in HM patients causes interstitial changes in the lung with more interlobular septal thickening, consistent with studies in Japanese HM patients (15). Clinically, patients in the true negative group had insignificant symptoms of infection. This may be attributed to the high percentage of DI-ILD in our study (40%, 6/15). One study showed that up to 1/3 of patients with DI-ILD did not exhibit symptoms such as cough and fever (16). Drugs that can cause DI-ILD include newer drugs such as pomalidomide (P14) (17), ponatinib (P13) (18) and traditional chemotherapeutic drugs such as cytarabine (19, 20). Patients’ pulmonary changes and symptoms were mostly closely associated with drug use; patients’ symptoms improved significantly after drug discontinuation, with DI-ILD receding to grade 1–2 (16). These findings suggest that when imaging in HM patients indicates significant interstitial lung changes and clinical symptoms of infection are not prominent, patients should be considered for possible non-infectious lung lesions related to the primary disease and treatment regimen. Physicians should retrace a patient’s prior medical history and analyze the patient’s treatment and medication history in conjunction with routine testing to reduce the number of high risk invasive tests performed in HM patients. However, due to the disease specificity of HM patients, a negative mNGS result can also yield important information. Some patients need to receive further immunosuppressive therapy (e.g., CAR T-cell therapy or HSCT), and a negative result can help physicians rule out potential infections, paving the way and reducing the risk from subsequent treatment. In addition, the low rate of antibiotic discontinuation in our true-negative patients is due to the fact that some patients still required antibiotic prophylaxis, especially those in a period of granulocyte deficiency (21).

A negative mNGS result does not completely exclude pulmonary infection, as mNGS analysis may be affected by multiple factors resulting in false-negatives. On the one hand, the detection of pathogens is influenced by the ratio of host nucleic acids, and high levels of host nucleic acids can reduce the detection of pathogens by mNGS (22, 23), and on the other hand, inefficient extraction of pathogenic nucleic acids or inspecting sites with pathogens below the positive threshold for detection can also result in false-negatives (24). Filtering strategies can also result in false-negatives (25). The five fungal pathogens and three cases of Mycobacterium tuberculosis overlooked during analysis in this study are considered thick-walled microorganisms. This can lead to increased difficulty in nucleic acid extraction (26, 27), a phenomenon corroborated in other studies. For bacteria, three of the four patients with bacterial infections not captured through mNGS returned results showing the presence of Gram-negative bacteria. Gram-negative bacteria and viruses may be degraded by lysis agents during pretreatment due to a lack of a protective shell and increased fragility, leading to DNA release and degradation and thereby resulting in decreased or even complete loss of their nucleic acid content (28). One patient, P26, had Pseudomonas maltophilia detected in analysis, but did not reach the positive threshold criteria. The above observations suggest that mNGS assays would benefit from continued optimization in sample processing and nucleic acid extraction. Additionally, analysis could be further improved by integrating bioinformatics and clinical knowledge.

What should clinicians do when encountering a negative mNGS result in a HM patient? First, clinicians should aim to fully integrate clinical observations, imaging and other tests to determine whether the result is a true negative. If there are other factors causing lung imaging changes, the clinician should analyze the patient’s medical history, primary disease, and treatment regimen to make targeted therapeutic adjustments. Second, if infection is still highly suspected, samples from different sites (from tissues and body fluids) can be sent for mNGS. In two tuberculosis (TB) patients in our study, the pathogen was eventually detected after mNGS analysis was conducted on pleural fluid and liver tissue; some studies suggest that the use of tissue and body fluid mNGS may be better than BALF mNGS for the detection of Mycobacterium tuberculosis (29). Third, CMTs can be repeated after a negative mNGS result. The final pathogen in 57.1% (8/14) of our false-negative patients was eventually detected through conventional microbiological tests, suggesting that a full work-up of CMTs is not necessarily inferior to mNGS (30). Fourth, clinicians should focus more on pathogens such as tuberculosis, fungi, and other microorganisms that are harmful to HM patients after failed mNGS test.

The value of mNGS results for clinical guidance of treatment has been reported in other studies (31). mNGS positive results guided the use of antibiotics in most patients but missed pathogen detection led to delays in treatment. Our analysis showed that for mNGS results that failed to detect Mycobacterium tuberculosis, patients experienced a much longer delay in receiving appropriate anti-tuberculosis treatment compared to patients whose mNGS results failed to detect bacterial infections (13 day vs. 1.5 day *P* < 0.05). Some studies suggest that delayed treatment of TB is associated with all-cause mortality (32), and that the risk of delayed diagnosis of TB is also higher in HM patients than in other immunocompromised populations (33). Furthermore, delayed diagnosis of leads to potential TB dissemination; screening for TB in mNGS-negative HM patients should be conducted whenever possible. For patients with fungal infections, recent studies suggest that preemptive antifungal therapy in immunocompromised patients reduces the overall duration and use of antifungal drugs and increases the detection rate of fungal infections compared with empirical therapy (34). Empirical therapy should be considered in patients with suspected fungal infections taking into context the patient’s condition.

Our study of negative mNGS results in alveolar lavage fluid from HM patients with suspected pulmonary infections has the potential to strengthen clinical guidance, but has some limitations. First, this study did not fully identify the specific pathogens detected in all false-negative patients. Second, this is a single-center retrospective study with a small sample size and selection bias. Results should be further validated in a large-scale, multicenter study. However, our study can help clinicians to a certain extent to correctly select HM patients who need BALF mNGS, reduce unnecessary invasive operations, and also provide a reference for physicians to improve diagnosis and treatment when faced with negative BALF mNGS results in HM patients, a population with high risk of lung infection and rapid post-infection progression.

## Data availability statement

The data presented in the study are deposited in the SRA repository, with the accession number PRJNA984933.

## Ethics statement

The studies involving human participants were reviewed and approved by The Institutional Review Board of the Third Xiangya Hospital, Central South University. Written informed consent from the participants’ legal guardian/next of kin was not required to participate in this study in accordance with the national legislation and the institutional requirements.

## Author contributions

ZD and YST performed this study and wrote the manuscript. QC and JZ revised this manuscript. YXT, ML, and FL participated in the collecting data and contributed to the data analysis. XL designed and directed the entire study. All the authors reviewed the final version of the manuscript.

## Footnotes

1
ftp://ftp.ncbi.nlm.nih.gov/genomes

2
https://www.hopkinsguides.com/hopkins/index/JohnsHopkinsABXGuide/Pathogens

3
https://www.clinmicronow.org/doi/book/10.1128/9781683670438.MCM
